# Functional Analysis of *FoCrpA* in *Fusarium oxysporum* Causing Rice Seedling Blight

**DOI:** 10.3390/jof11040317

**Published:** 2025-04-17

**Authors:** Chun Wang, Liang Wang, Xuanjie Zhao, Lei Hou, Qingran Liu, Rui Ren, Anqi Lv, Xinyang Liu, Tianliang Xiong, Peng Guo, Xiaofeng Xu, Zhe Ni, Chunlai Liu, Junhua Zhang

**Affiliations:** 1 College of Plant Protection, Northeast Agricultural University, Harbin 150030, China; chunharbin@aliyun.com (C.W.);; 2 Institute of Plant Protection, Heilongjiang Academy of Agricultural Sciences/Scientific Observing and Experimental Station of Crop Pests in Harbin, Ministry of Agriculture and Rural Affairs, Harbin 150086, China

**Keywords:** *Fusarium oxysporum*, rice seedling blight, copper-transporting phosphorylating ATPase, pathogenicity

## Abstract

*Fusarium oxysporum* is one of the main pathogens causing rice seedling blight disease. Revealing its pathogenic mechanism is of great significance for formulating prevention and control strategies for rice seedling blight disease. Copper transporting P-type ATPases (Cu-ATPase) is a large class of proteins located on the plasma membrane that utilize the energy provided by ATP hydrolysis phosphorylation to transport substrates across the membrane. It plays a crucial role in signal transduction, the maintenance of cell membrane stability, and material transport. The main function of Cu-ATPase is to maintain the homeostasis of copper in cells, which is essential for the normal growth and development of organisms. This study utilized the ATMT-mediated gene knockout method to obtain the knockout mutant *∆FoCrpA* and the complementation strain *∆FoCrpA-C*, which are highly homologous to the P-type heavy metal transport ATPase family in *F. oxysporum*. The results showed that, compared with the wild-type strain, the knockout mutant *∆FoCrpA* had a lighter colony color; a reduced tolerance to copper ion, osmotic, and oxidative stress; a weakened ability to penetrate glass paper; and decreased pathogenicity. However, there was no significant difference in pathogenicity and other biological phenotypes between the complementation strain *∆FoCrpA-C* and the wild-type strain. In summary, the *FoCrpA* gene is involved in osmotic and oxidative stress, affecting the invasion and penetration ability and pathogenicity of *F. oxysporum*, laying a theoretical foundation for understanding the development and pathogenic mechanism of *F. oxysporum*.

## 1. Introduction

Rice seedling blight poses a significant threat to global rice production, resulting in substantial losses in yield and quality [1,2]. This disease, which is among the most important rice seedling diseases, is caused by a diverse array of pathogens, including *Fusarium* spp. [3,4], *Burkholderia plantarii* [5], *Rhizoctonia solani* [6], *Marasmius graminum* [7], *Pythium aristosporum* [8], and *Cochliobolus carbonum* [9]. In Northeast China, *Fusarium oxysporum* has emerged as the predominant pathogen responsible for rice seedling blight [10]. Given the scarcity of effective resistant rice varieties, chemical fungicides have become one of the most widely used methods for controlling this disease [11]. However, the prolonged application of chemical fungicides has led to the development of drug resistance in *F. oxysporum* [12,13,14,15]. Consequently, investigating the functions of pathogenesis-related genes in *F. oxysporum* is essential for elucidating its pathogenic mechanisms and developing sustainable control strategies.

Copper is an essential micronutrient for the normal physiological activities of living organisms. It serves as a component and active site in various redox reaction proteins, such as superoxide dismutase and cytochrome C oxidase, and is involved in numerous biological processes and metabolic activities, including photosynthesis, electron transfer, protein synthesis, and the alleviation of oxidative stress [16]. Copper ions play a crucial role in maintaining the metabolism and normal development of organisms [17]. Both copper deficiency and excess can be detrimental; copper ion deficiency can impair the activity of enzymes that rely on copper ions as a cofactor, thereby affecting cell survival [18]. In contrast, excessive copper ions can cause lipid and protein peroxidation, generating large amounts of reactive oxygen species that disrupt cell structure, impair cell division, and damage enzymatic systems [19]. Additionally, excess copper ions can react with cellular thiols, displacing other metals in proteins and causing oxidative damage at the nucleic acid level [20]. Cu-ATPase, a large class of membrane proteins located on the plasma membrane, utilizes the energy from ATP hydrolysis to transport substrates across the membrane. It plays a vital role in signal transduction, maintaining cell membrane stability, and facilitating substance transport [21]. Cu-ATPase comprises five subfamilies that transport different substrates. Among them, the P1B-type ATPase (Heavy Metal ATPase, HMA) is a subfamily of P-type ATPase that selectively absorbs and transports essential metal ions such as Cu^+^, Cu^2+^, Zn^2+^, Co^2+^, and Cd^2+^ [22]. The primary function of Cu-ATPase in HMA is to maintain cellular copper homeostasis, which is critical for normal growth and development [23]. In some pathogenic fungi, Cu-ATPase is required for normal growth, reproduction, and effective host invasion. For instance, in the filamentous plant pathogen *Cochliobolus heterospira*, mutation of the *ChCcc2* gene significantly affects its growth rate, pigmentation, spore count, and colony morphology [24]. In the pathogen *Colletotrichum lindemuthianum*, a knockout mutant of the *clap1* gene exhibited similar nutritional growth and spore formation as the wild type but displayed lighter pigmentation, fewer appressoria, and no disease symptoms [25]. In the ubiquitous fungus *Aspergillus flavus,* the deletion of *CrpA* and *CrpB* resulted in reduced toxicity to mice and a diminished colonization ability on corn seeds treated with copper fungicides [26]. Despite these findings, the role of Cu-ATPase in the pathogenicity of *F. oxysporum* remains poorly understood, necessitating further investigation into its specific mechanisms and impact on virulence. 

Previously, we identified multiple pathogenesis-related genes, including *FoCrpA*, using a T-DNA insertion mutant library of the rice seedling blight fungus *F. oxysporum*. Through integrated bioinformatics characterization of the *FoCrpA*-encoded protein and functional validation via *Agrobacterium tumefasciens*-mediated transformation (ATMT)-based homologous recombination, this study systematically investigated the biological role of *FoCrpA* in *F. oxysporum*. Our approach enabled comprehensive evaluation of *FoCrpA* influence on fungal adaptive mechanisms, particularly focusing on stress response pathways and virulence regulation. These findings advance our understanding of *F. oxysporum* pathogenicity determinants, reveal promising molecular targets for next-generation antifungal agents, and provide theoretical foundation for developing novel disease management strategies against rice seedling blight.

## 2. Materials and Methods

### 2.1. Fungal Strains, Rice Variety, Plasmids

The *F. oxysporum* wild-type strain Fo21, originally isolated from symptomatic rice seedlings exhibiting rice seedling blight in Heilongjiang Province, China, served as the progenitor for genetic manipulation. All fungal strains including WT Fo21, *∆FoCrpA* mutants, and complemented strain *∆FoCrpA-C* were maintained on potato dextrose agar (PDA: 200 g/L potato, 20 g/L glucose, 15 g/L agar). The rice cultivar Longjing 31 (*O. sativa* subsp. *japonica*), provided as pathologically certified seeds by the Phytopathology Laboratory of Northeast Agricultural University (Harbin, China). *Agrobacterium tumefaciens* AGL-1 was gifted by Professor Zhang Shihong of Jilin University. *Escherichia coli* DH5α was gifted by Professor Qin Qingming of Jilin University. The plasmid pXEH was gifted by Professor Li Guihua from Jilin University, which contains the hygromycin B resistance gene and is used for gene knockout. The plasmid pSUL was gifted by Professor Zhang Shihong from Jilin University, which contains the resistance gene Chlorpyriprone and is used for gene complementation.

### 2.2. Protein Sequence Analysis and Phylogenetic Reconstruction

The coding sequence of *FoCrpA* (GenBank accession: XP_018237293.1) was retrieved from the NCBI RefSeq database. Functional domain architecture was resolved through complementary analyses usingNCBI Conserved Domain Database (CDD) and SMART. Orthologous *CrpA* sequences from 15 taxonomically diverse Ascomycete fungi were downloaded from GenBank. Multiple sequence alignment and the maximum-likelihood phylogenetic tree was reconstructed in MEGA7.0 with 1000 bootstrap replications.

### 2.3. Targeted Gene Disruption and Complementation

Gene knockout and complementation were performed via *Agrobacterium tumefaciens*-mediated transformation (ATMT) using the binary vector system [27,28]. *FoCrpA* disruption mutant was generated by amplifying upstream and downstream flanking regions of *FoCrpA* from wild-type Fo21 genomic DNA using primers FoCrpA-UP-F/R and FoCrpA-DN-F/R. The plasmid pXEH was linearized with *EcoRI,* and the upstream fragment was ligated into the *EcoRI* site via ligation-independent cloning (Takara Bio). Recombinant plasmid pXEH-CUP was transformed into *E. coli* DH5α and validated by colony PCR (primers FoCrpA-UP-F/Hup-R). The downstream fragment was subsequently inserted into the *SalI* site of pXEH-CUP, yielding the final knockout vector pXEH-C. Following *Agrobacterium tumefaciens* AGL-1-mediated transformation with Fo21 conidia (1 × 10^6^ spores/mL), transformants were selected on PDA containing 80 µg/mL hygromycin B, 300 µg/mL carbenicillin, and 150 µg/mL cefotaxime. Putative mutants were subcultured thrice and validated via multiplex PCR: HPH integration (Hyg-F/R, 1.2 kb), homologous recombination junctions (Cup-F/Hup-R: 800 bp; Hdn-F/Cdn-R: 750 bp), and *FoCrpA* knockout confirmation (FoCrpA-F/R, no amplification).

The complementation fragment C-*FoCrpA* (4880 bp), encompassing the full-length *FoCrpA* gene, was amplified using primers C-FoCrpA-F/R and purified. Plasmid pSUL was digested with *BamHI*, and the C-*FoCrpA* fragment was ligated into the *BamHI* site. The resultant complementation vector pSUL-C was validated through colony PCR, *BamHI* restriction digestion, and sequencing. pSUL-C was introduced into *Agrobacterium tumefaciens* AGL-1 and co-cultivated with *∆FoCrpA* conidia. Transformants were selected on chlorimuron-ethyl-supplemented medium and verified by PCR amplification using primers FoCrpA-F/R. Successfully complemented strains, designated *∆FoCrpA-C*, were confirmed by restored *FoCrpA* amplification. Mutants were verified by PCR and qRT-PCR. PCR amplification was performed in a 10 μL reaction volume containing: 5 µL 2 × Taq PCR Master Mix, 0.4 µL *FoCrpA*-F, 0.4 µL *FoCrpA*-R, 0.4 µL strains DNA, 3.8 µL ddH_2_O to adjust the final volume. PCR (Bio-rad T100, Hercules, CA, USA) amplification was conducted under the following thermocycling conditions: initial denaturation at 94 °C for 5 min, followed by 30 cycles of denaturation (94 °C, 30 s), annealing (55 °C, 30 s), and extension (72 °C, 1 min), with a final elongation step at 72 °C for 10 min. The ChamQ Universal SYBR qPCR Master Mix (Vazyme, Nanjing, China) was used to perform quantitative real-time PCR (qRT-PCR), and qRT-PCR assays ran on a QuantStudio^TM^5 real-time PCR system. qRT-PCR reaction system was performed in a 20 μL reaction volume containing: 2xFast qPCR Master Mixture(Green) 10 µL, Actin-F/*FoCrpA*-F 0.5 µL, Actin-R/*FoCrpA*-R 0.5 µL, DNA template 1 µL, DEPC-ddH_2_O 8 µL. qPCR amplification was conducted under the following thermocycling conditions: initial denaturation at 94 °C for 2 min, followed by 40 cycles of denaturation (94 °C, 15 s), annealing (60 °C, 15 s), and extension (72 °C, 45 s). Data analysis was carried out using the delta delta-CT (2^−ΔΔCt^) method outlined by Livak and Schmittgen [29]. The primer sequences used are shown in Table 1.

### 2.4. Phenotype Analysis

Mycelial plugs (5 mm diameter) from Fo21, *∆FoCrpA*, and *∆FoCrpA-C* were placed on PDA plates. For colony morphology, plates were incubated in the dark at 25 °C for 7 days, and then colony diameter and morphology were measured [11]. For mycelial dry weight and culture color, mycelial plugs were inoculated into 100 mL PDB medium and incubated at 180 rpm at 25 °C for 2 days. Culture color was observed, and hyphae were collected by centrifugation at 10,000 rpm for 15 min, dried at 80 °C to constant weight, and weighed. For copper ion stress, mycelial plugs were incubated on PDA plates with 0 mM, 0.25 mM, 0.5 mM, and 1 mM CuSO_4_ in the dark at 25 °C for 5 days. For conidial production and germination, mycelial plugs were inoculated into 50 mL PDB medium incubated at 180 rpm at 25 °C for 2 days. Then, 100 µL suspension (1 × 10^8^ spores/mL) was inoculated into 40 mL germination medium (GM: 20 g Sucrose, 1 g NaNO_3_, 0.5 g KH_2_PO_4_, 0.5 g NaCl, and 0.5 g MgSO_4_ in 1 L of distilled water) in a shaker at 180 rpm at 25 °C for 6 h, 9 h, and 12 h to calculate percentage of conidial germination. For stress response, mycelial plugs were placed on PDA plates amended with 1 M sorbitol, 0.5 M NaCl, 0.03% SDS, 1 mM Congo red, and 10 mM H_2_O_2_ and incubated in the dark at 25 °C for 5 days. For penetration capacity evaluation, cellophane membranes were overlaid on PDA plates. Post 48 h colonization, membranes were aseptically removed to assess substrate penetration through residual colony development during extended incubation (24 h). The colony diameters were measured after being incubated and the inhibition rates of various stress factors were calculated. Each experiment was repeated three times.

### 2.5. Toxicity Assays with Culture Filtrates and Pathogenicity Assay 

Next, 5 mm mycelial plugs from Fo21, *∆FoCrpA*, and *∆FoCrpA-C* strains were cultured in 100 mL PDB medium (25 °C, 100 rpm, 7 d). The culture was filtered, centrifuged (4000 rpm, 15 min), and the supernatant filtered through 0.22 µm membranes to obtain crude toxin extract. The surface-sterilized (70% ethanol, immersed for 1 min) rice seeds were incubated with the extract (PDB medium as control) under 25 °C light for 5 d. Germination rate and shoot lengths were determined using three biological replicates, each containing fifty rice seeds. For germination assessment, seeds with radicle emergence ≥ 2 mm were considered germinated. Shoot length was measured from coleoptile base to tip using digital calipers.

Rice seeds were surface-sterilized with 70% ethanol for 1 min, rinsed 3 times with sterile water, and subjected to germination pretreatment on sterile filter paper at 25 °C for 2 days. Spore suspensions of Fo21, *∆FoCrpA*, and *∆FoCrpA-C* strain were prepared, and conidial concentrations were adjusted to 1 × 10^6^ conidia/mL. Germinated seeds were soaked in the conidial suspensions for 30 min, with sterile water as the control, then sown in pots on top of potting soil (vermiculite/soil ratio of 1:2) and covered with autoclaved sterile soil. Disease progression was observed after 14 days of incubation at 25 °C. Each treatment consisted of 50 rice plants with three replicates. Disease index was calculated based on rice seedling blight grading standards from previous research using a scale from 0 to 4, defined as follows [30]: 0 = no symptoms; 1 = a few small lesions below 1/4 of the stem circumference; 2 = large lesions occupying 1/4–1/2 of stem circumference; 3 = large lesions occupying 1/2–3/4 of stem circumference; 4 = lesions occupying all stem circumference and plant die. The disease index was calculated as follows: ∑(No.of diseased plants × relative grade) × 100/(total No. of investigated plants × the highest grade).

### 2.6. Statistical Analysis

All experiments were performed at least thrice using independent assays. One-way analysis of variance (ANOVA) was conducted using SPSS (version 22.0 for windows, 2013, IBM, Armonk, NY, USA). The statistical significance of data comparisons was performed between the wild type Fo21 and the deletion mutants with one-way analysis of variance (ANOVA), followed by Duncan’s multiple range test. Values of *p* < 0.05 were labeled as statistically significant.

## 3. Results

### 3.1. Identification of FoCrpA from F. oxysporum

**Figure 1:**
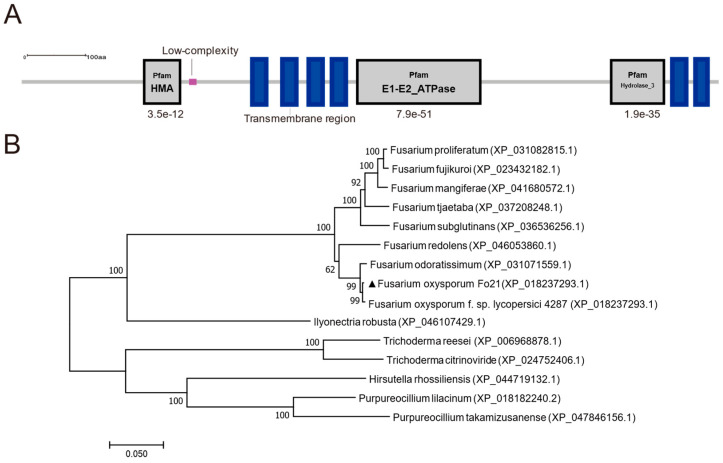
Functional domain architecture and phylogenetic analysis of *FoCrpA* in *F*. *oxysporum*. (**A**) Domain prediction of FoCrpA via the SMART database identified three conserved motifs: 1. Pfam: HMA (Heavy Metal-Associated) domain (aa 45–86): A β-sandwich fold critical for heavy metal ion binding and transport, conserved across prokaryotic and eukaryotic metal transporters. 2. Pfam: E1-E2_ATPase (aa 210–450): Characteristic of P-type ATPases, featuring an ATP hydrolysis module (E1, aa 210–320) for energy release and a transmembrane ion translocation module (E2, aa 350–450). 3. Pfam: Hydrolase_3 (HAD-like) (aa 500–620): A haloacid dehalogenase superfamily domain catalyzing phosphatase/hydrolase reactions, potentially linked to metal cofactor metabolism. (**B**) Maximum-likelihood phylogenetic tree of *FoCrpA* homologs across 9 *Fusarium* species and 6 other fungal species, reconstructed with MEGA7.0 (1000 bootstrap replicates).

Bioinformatic analysis of *FoCrpA* using the NCBI Conserved Domain Database (CDD) identified its C-terminal region (residues 371–1081) as a canonical member of the P-type ATPase superfamily (PF00122), specifically classified as a heavy metal-transporting ATPase (HM-ATPase). Subsequent SMART analysis further resolved three conserved functional modules: (i) N-terminal HMA domain (PF00403, aa 45–86): A ββαβ metal-binding fold critical for heavy metal ion recognition and chelation. (ii) Central E1-E2_ATPase domain (PF00122, aa 210–450): Characteristic catalytic core of P-type ATPases, comprising E1 ATP-binding subdomain (aa 210–320) with conserved DKTGT phosphorylation motif and E2 transmembrane ion translocation subdomain (aa 350–450) containing CPC metal coordination residues; (iii) C-terminal Hydrolase_3 domain (PF13343, aa 500–620): A HAD-family phosphatase module potentially regulating ATPase autoinhibition (Figure 1A). Phylogenetic reconstruction using maximum-likelihood methods (MEGA7.0) of 15 ascomycete orthologs revealed strong clustering of *F. oxysporum FoCrpA* with *F. oxysporum* f. sp. *lycopersici* (XP_018237293.1, 99% identity), forming a distinct clade from *Fusarium odoratissimum* (XP_031071559.1) and *Fusarium redolens* (XP_046053860.1), while the protein from other fungi displays low similarity with homologous proteins (Figure 1B). This phyletic pattern suggests vertical inheritance of *FoCrpA* within the *Fusarium* genus, with functional conservation in metal homeostasis.

### 3.2. Generation of FoCrpA Deletion Mutants and Complementation Assay

**Figure 2:**
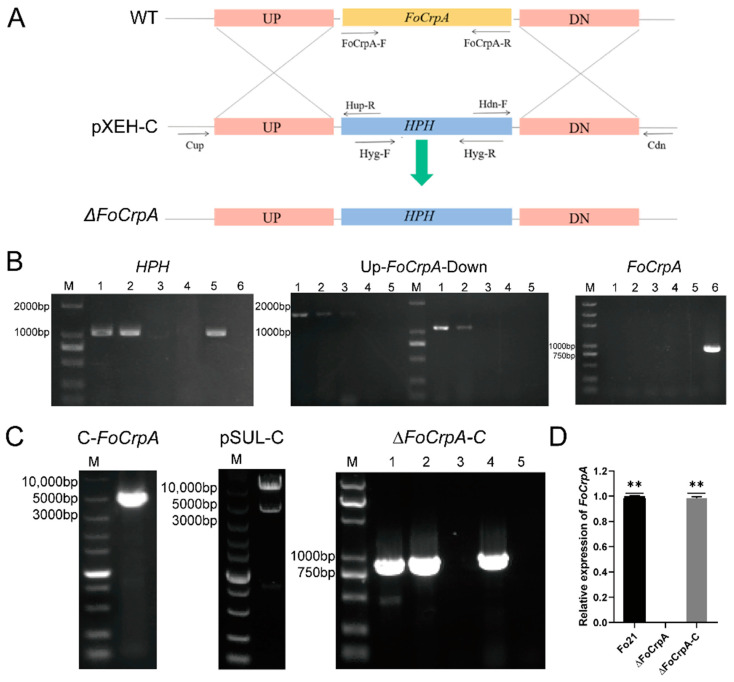
*FoCrpA* gene knockout strategy; PCR and qRT-PCR validation of knockout mutants and complementation strains. (**A**) *FoCrpA* gene knockout strategy. (**B**) PCR validation of *HPH* gene (M: DL2000 Ladder DNA Marker; 1–3: Mutant; 4: Fo21; 5: pXEH; 6: ddH_2_O); PCR validation of the two side fragments of *FoCrpA* gene (M: DL2000 Ladder DNA Marker; 1–3: mutant; 4: Fo21; 5: ddH_2_O); PCR validation of *FoCrpA* gene (M: DL5000 Ladder DNA Marker; 1–3: Mutant; 4: pXEH; 5: ddH_2_O; 6: Fo21). (**C**) C-*FoCrpA* gene fragment (M: 1 kb Ladder DNA Marker); pSUL-C enzyme digestion validation (M: 1 kb Ladder DNA Marker); PCR validation of *∆FoCrpA*-C (M: DL5000 Ladder DNA Marker; 1–2: complementation mutant; 3: *∆FoCrpA*; 4: Fo21; 5: ddH_2_O). (**D**) Expression analysis of *FoCrpA* in WT Fo21 and its mutants strains with qRT-PCR. Duncan’s multiple range test was performed to determine significant difference, ** *p* ≤ 0.01.

*FoCrpA* was deleted using a homologous recombination strategy (Figure 2A). The gene knockout vector pXEH-C was transferred into *Agrobacterium tumefaciens* AGL-1 to transform the wild-type Fo21 strain. The *FoCrpA* gene in *F. oxysporum* was replaced with the hygromycin resistance gene via homologous recombination (Figure 2A). Four primer pairs (Hyg-F/Hyg-R, Cup-Hup-R, Hdn-F/Cdn, *FoCrpA*-F/*FoCrpA*-R) were used to validate the knockout mutant over three generations (Figure 2B). The results showed that two mutants successfully integrated the hygromycin resistance gene (detected by Hyg-F/Hyg-R). The upstream (1531 bp) and downstream (1102 bp) fragments of *FoCrpA* were correctly integrated in two mutants (validated by Cup-Hup-R and Hdn-F/Cdn). Three mutants lacked the *FoCrpA* gene (confirmed by *FoCrpA*-F/*FoCrpA*-R). These results confirmed the successful knockout of the *FoCrpA* gene, and the mutant was named *∆FoCrpA*.The *FoCrpA* locus (4880 bp) was cloned from Fo21 into pSUL via *BamHI,* generating complementation vector pSUL-C (PCR/restriction-verified). *Agrobacterium tumefasciens*-mediated transformation of pSUL-C into *ΔFoCrpA* restored the target fragment (867 bp PCR-amplified in complemented strain *∆FoCrpA-C* and WT, absent in *∆FoCrpA*), confirming genetic complementation (Figure 2C). Transcriptional validation via qRT-PCR using the internal reference gene β-actin as a control demonstrated complete abolition of *FoCrpA* expression in *∆FoCrpA* (expression level <0.01% of wild-type Fo21, normalized to 1), with full transcriptional restoration observed in the complemented strain *∆FoCrpA-C* (Figure 2D). This indicates that *FoCrpA* was disrupted successfully in *ΔFoCrpA* and reintegrated in *ΔFoCrpA-C*.

### 3.3. Effects of FoCrpA on Vegetative Growth and Conidiogenesis

**Figure 3:**
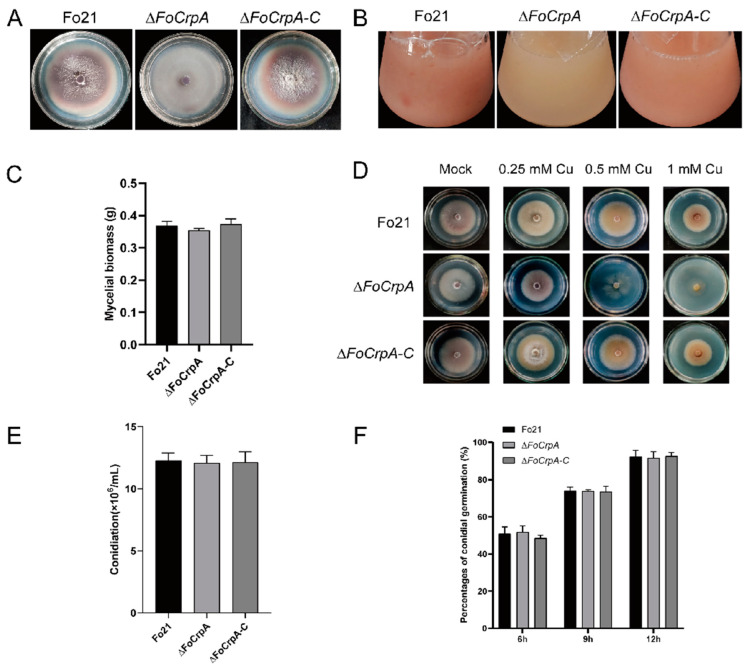
Phenotypic analysis of *FoCrpA* and its mutant strains. (**A**) Colony phenotypic characterization of Fo21, *∆FoCrpA*, and *∆FoCrpA-C* on PDA plates after incubation of 7 days at 25 °C. (**B**) Observing culture color of Fo21, *∆FoCrpA*, and *∆FoCrpA-C* strains grown in 100 mL PDB in a shaker at 180 rpm at 25 °C for 2 days. (**C**) Mycelial biomass was measured by determining the dry weight of Fo21, *∆FoCrpA*, and *∆FoCrpA-C* strains grown in 100 mL PDB in a shaker at 180 rpm at 25 °C for 3 days. (**D**) Colony morphology of Fo21, *∆FoCrpA*, and *∆FoCrpA-C* under Cu stress, amended with CuSO_4_ at different concentrations indicated in the figure and cultured at 25 °C for 5 days. (**E**) Conidial production of Fo21, *∆FoCrpA* and *∆FoCrpA-C* grown in 50 mL PDB in a shaker at 180 rpm at 25 °C for 2 days. (**F**) Conidial germination was compared between Fo21 and its mutants by re-suspending the conidia in 40 mL liquid GM in a shaker at 180 rpm at 25 °C for 6 h, 9 h, and 12 h.

Colony phenotypic characterization of *FoCrpA* and its mutant strains demonstrated that wild-type Fo21 and its mutant strains exhibited similar spreading growth patterns with comparable radial expansion rates, while *∆FoCrpA* colonies displayed significant pigmentation attenuation compared to the wild-type Fo21 and complemented strains, which showed indistinguishable colony coloration (Figure 3A). For the color of the liquid culture, *∆FoCrpA* also produced visibly lighter extracellular pigments than wild-type and complemented strains (Figure 3B). Liquid cultures grown in PDB medium revealed no significant differences in mycelial biomass accumulation among strains (Figure 3C), demonstrating that *FoCrpA* deletion does not impair hyphal growth under standard nutrient conditions. Tolerance assays on CuSO_4_-supplemented PDA (0–1 mM) demonstrated that *∆FoCrpA* exhibited significant growth suppression at 0.5 mM and complete growth arrest at 1 mM CuSO_4_, whereas wild-type and complemented strains maintained partial viability (Figure 3D). Conidial production and germination were similar between WT Fo21 and its mutant strains (Figure 3E,F). These results demonstrate that *FoCrpA* specifically modulates pigment biosynthesis and copper ion homeostasis in *F. oxysporum*, while exhibiting no significant regulatory effects on mycelial biomass accumulation, conidiogenesis, or conidial germination.

### 3.4. Response of FoCrpA to Stress Agents

**Figure 4:**
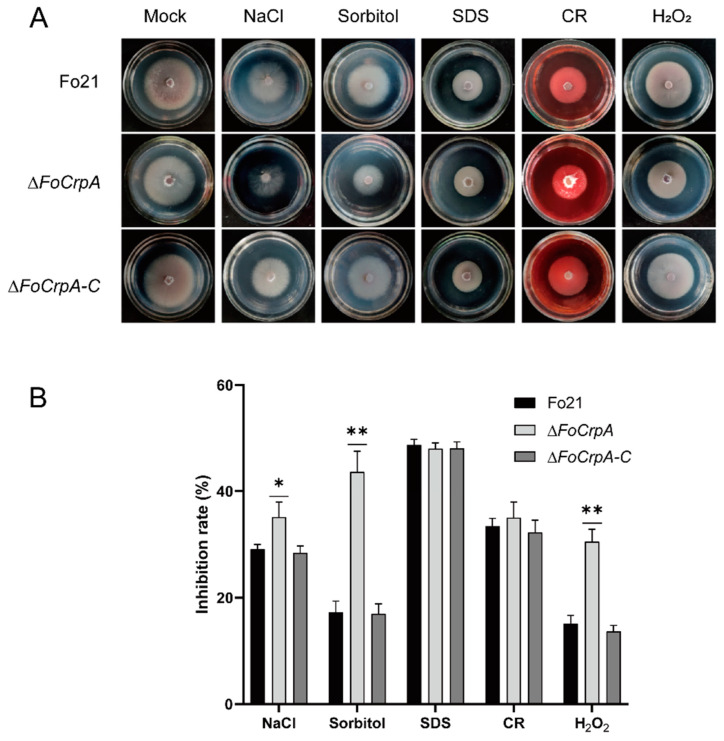
Deletion of *FoCrpA* affects various stress response. (**A**) Colony growth of Fo21, *∆FoCrpA*, and *∆FoCrpA-C* strains on PDA plates supplied with 0.5 M NaCl, 1 M sorbitol, 0.03% SDS, 1 mM Congo red, or 10 mM H_2_O_2_ after incubation of 5 days at 25 °C. (**B**) Inhibition rate of colony growth by different stress factors. Error bars represent the standard error of mean. Duncan’s multiple range test was performed to determine significant difference,* *p* ≤ 0.05,** *p* ≤ 0.01.

To investigate the regulatory role of *FoCrpA* in environmental stress adaptation of *F. oxysporum*, comparative phenotypic analyses were performed using wild-type Fo21, *∆FoCrpA*, and *∆FoCrpA*-C on PDA plates supplemented with different stress agents, including NaCl and sorbitol (osmotic stresses), SDS and CR (cell wall stresses), and H_2_O_2_ (oxidative stress). After five-day incubation at 25 °C, the *∆FoCrpA* mutant displayed significantly impaired growth under osmotic and oxidative stress conditions compared to the wild-type Fo21 and complemented strain *∆FoCrpA*-C, while maintaining wild-type-level sensitivity to cell wall stressors (Figure 4A,B). These data collectively demonstrate that *FoCrpA* specifically modulates *F. oxysporum*’s tolerance mechanisms against osmotic and oxidative challenges. 

### 3.5. Culture Filtrates Toxicity from FoCrpA Mutants

**Figure 5:**
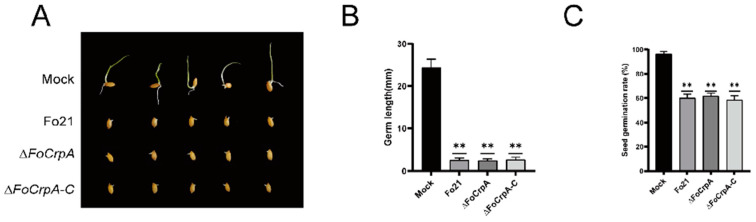
Toxicity of culture filtrates from Fo21 and *FoCrpA* mutants on the growth of rice seeds. (**A**) Culture filtrates of Fo21, *∆FoCrpA*, and *∆FoCrpA-C* strains caused defects in rice seeds growing. PDB medium without culture filtrates was used as the blank control. (**B**) Differences of germ length were examined after incubation under light at 25 °C for 5 days. (**C**) Seed germination rate was compared between WT Fo21 and its mutants after incubation under light at 25 °C for 5 days. Error bars represent the standard error of mean. Duncan’s multiple range test was performed to determine significant difference,** *p* ≤ 0.01.

Toxicity assessment using culture filtrates from Fo21 and its mutants revealed significant suppression of rice seed germination and germ length compared to the PDB blank control (Figure 5A). Notably, no statistically significant differences in these germination parameters were observed among filtrate-treated groups (Figure 5B,C). These findings demonstrate that *F. oxysporum* secretes germination-inhibitory metabolites into the extracellular milieu, while crucially establishing that *FoCrpA* does not participate in the biosynthesis or secretion of these toxic compounds.

### 3.6. Effect of FoCrpA Mutants on Pathogenicity

**Figure 6:**
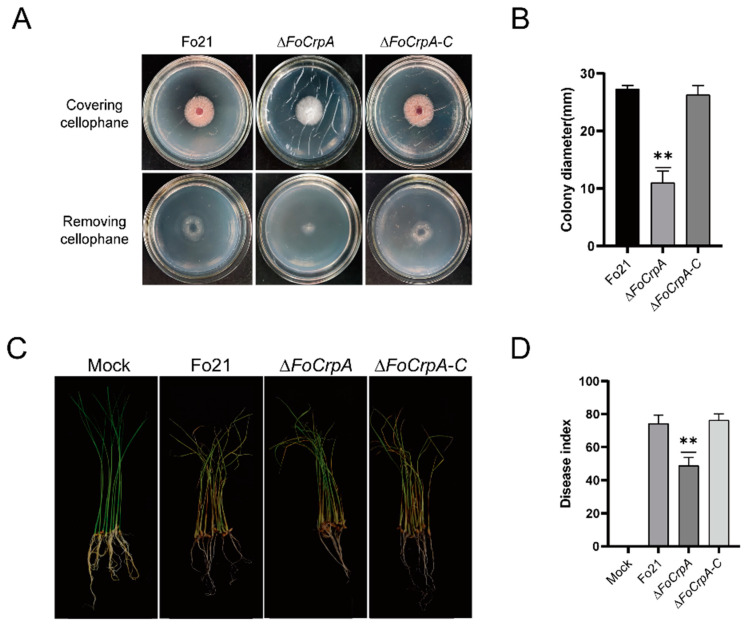
Pathogenicity of Fo21 and *FoCrpA* mutants on the stem base of rice seedlings. (**A**) Comparison of infection–penetration morphology caused by inoculation with Fo21, *∆FoCrpA*, and *∆FoCrpA-C* strains on cellophane membranes after 3 days of incubation at 25 °C. (**B**) Differences of colony diameter incubating 1 day at 25 °C after removing cellophane. (**C**) Disease symptoms on the stem base of rice seedlings observed at 14 dpi wtih Fo21, *∆FoCrpA*, and *∆FoCrpA-C* strains. (**D**) Disease index of rice seedlings was determined at 14 dpi wtih Fo21, *∆FoCrpA*, and *∆FoCrpA-C* strains. Fifty rice seedlings were inoculated per replication. Error bars represent the standard error of mean. Duncan’s multiple range test was performed to determine significant difference,** *p* ≤ 0.01.

The infection–penetration assay conducted with Fo21, *∆FoCrpA*, and *∆FoCrpA*-C strains demonstrated comparable growth patterns across all strains when cultured on PDA plates overlaid with cellophane membranes. However, upon removal of the cellophane barrier followed by 24 h incubation, Fo21 and *∆FoCrpA*-C strains exhibited normal tissue penetration capabilities, and *∆FoCrpA* displayed significantly impaired colonization efficiency, as evidenced by reduced colony diameter (Figure 6A). This phenotypic divergence strongly suggests that *FoCrpA* plays a critical role in mediating host tissue invasion and penetration processes in *F. oxysporum*.

Pathogenicity assay results showed that most of the stems of rice seedlings inoculated with Fo21 and *∆FoCrpA*-C strain turned brown, and some leaves turned yellow and wilted with a disease index of 74.30 and 76.37, respectively (Table 2). However, rice seedlings inoculated with *∆FoCrpA* strain showed milder symptoms than those with Fo21 and *∆FoCrpA*-C (Figure 6B,C). The results suggest that the deletion of *FoCrpA* reduces the pathogenicity of *F. oxysporum*.

## 4. Discussion

Copper performs a comprehensive and vital function in biological systems. It orchestrates the conformational and catalytic properties of numerous metalloproteins and enzymes, as typified by cytochrome oxidase and superoxide dismutase. Additionally, due to the propensity of copper to react with non-specific proteins and engender toxic manifestations, a highly intricate and elaborate system is requisite for the accomplishment of processes such as its assimilation, concentration regulation, translocation to specific protein-binding sites, and expulsion. This complex assemblage comprises small carriers, molecular chaperones, and active transporters [31]. From our established mutant library of *F. oxysporum*, a Cu-ATPase belonging to the HMA subfamily was discovered. It is speculated that it plays a role in the pathogenic process of *F. oxysporum*. The functional domain analysis of the encoded protein showed that the *FoCrpA* gene encoded protein belongs to the P-type heavy metal transport ATPase family, which is located on the plasma membrane. In *Aspergillus nidulans*, gene *CrpA* coordinates the transport of copper ions between the plasma membrane, endoplasmic reticulum, Golgi apparatus, and other organelles [32]. In this study, by analyzing the biological functions of wild-type strain Fo21, knockout mutant *∆FoCrpA*, and complementation mutant *∆FoCrpA*-C, we investigated the function of the *FoCrpA* gene in *F. oxysporum*. It was found that *∆FoCrpA* had no significant differences in mycelial dry weight, spore production, spore germination rate, and tolerance to cell membrane stress and cell wall stress compared to wild-type strains. However, compared with the wild-type strain, the colony and liquid color of *∆FoCrpA* and its tolerance to high osmotic stress and oxidative stress decreased.

Cu-ATPase is involved in regulating the dynamic balance of copper metabolism, and copper is a cofactor for various copper-containing proteins, such as Cu/Zn superoxide dismutase (Cu/Zn SOD), laccase, etc. [33,34]. These enzymes are key enzymes in many biological reactions, such as Cu/Zn SOD, which can catalyze the decomposition of superoxide anions (O^2-^) into less toxic hydrogen peroxide [35,36]. When pathogens invade host plants, it helps them resist the extracellular ROS produced by the host plants [37,38,39]. Moreover, laccase is involved in various biological processes such as fungal morphogenesis, pathogen infection, pigment synthesis, and stress response [40]. In this study, we found that compared with the wild-type strain Fo21, the colony and liquid color of the knockout mutant *∆FoCrpA* and its tolerance to high osmotic stress and oxidative stress decreased, indicating that the *FoCrpA* gene can affect the pigment accumulation of *F. oxysporum* and its tolerance to exogenous osmotic stress and oxidative stress. Together with previous studies performed in other pathogenic fungi, such as in *Verticillium dahliae,* the knockout mutant *ΔVdSOD1* of the Cu/Zn *SOD* gene has reduced tolerance to oxidative stress [41]. In *Colletotrichum gloeosporiides,* the colony color of deletion mutant *ΔA2LAC1* changed from gray-black to nearly white, significantly reducing its pathogenicity to mangoes [42]. These results indicated that Cu-ATPase and other copper-containing proteins play a significant role in the growth and development of plant pathogenic fungi.

Our study also found that the deletion of the *FoCrpA* gene can lead to a decrease in the penetration ability of *F. oxysporum*, indicating that the *FoCrpA* gene plays an important regulatory role in the penetration ability of *F. oxysporum*. When *F. oxysporum* infects colonized host plants, it produces infection structures such as infection mats and attachment cells. In *Botrytis cinerea*, the deletion of the *BcCcc2* gene resulted in a diminished capacity to form attachment cells and infection mats, consequently impairing the fungus’s ability to invade plant epidermal tissues [43]. It is hypothesized that the functionality of infection structures, including infection pads and attachment cells, might depend on unidentified and essential copper-containing proteins. Cu-ATPase potentially modulates the activity of these proteins. Similarly, the deletion of the *FoCrpA* gene can reduce the pathogenicity of *F. oxysporum*, indicating that the *FoCrpA* gene may affect the pathogenicity of *F. oxysporum* by reducing its invasion and penetration ability.

Excessive intracellular copper ions can affect cell structure, leading to disruptions in cell division and enzymatic systems, thereby affecting fungal growth. Cu-ATPase can eliminate excess copper ions from the cell and alleviate the inhibitory effect of excessive copper ions on fungal growth. For example, after knocking out Cu-ATPase *CrpA* in *Aspergillus fumigatus*, the strain is more sensitive to high concentrations of copper ions [44]. After knocking out the *FgCrpA* gene of *F. graminearum*, the knockout mutant showed inhibition of hyphal growth, decreased spore production and germination rate, reduced tolerance to reactive oxygen species, and decreased production of toxin deoxynivalenol under copper ion stress compared to the wild type [45]. Therefore, this study conducted colony growth experiments under copper stress on wild-type strains, *∆FoCrpA* strains, and *∆FoCrpA*-C strains. It was found that compared with the wild-type, *∆FoCrpA* was more sensitive to copper ions, and the colony diameter of *∆FoCrpA* under the same copper ion concentration was smaller, and the mycelial growth was sparser. When copper ions increased to a certain concentration, *∆FoCrpA* almost did not grow, while the wild-type strain could continue to grow. The deletion of the *FoCrpA* gene can exacerbate the inhibitory effect of excessive copper ions on fungus growth.

Overall, the *FoCrpA* gene is involved in osmotic and oxidative stress, affecting the invasion and penetration ability and pathogenicity of *F. oxysporum*. The *FoCrpA* study identifies copper homeostasis as a key vulnerability in *F. oxysporum*. Targeting this system—via Cu-ATPase inhibitors, optimized copper fungicides, or host-enhanced ROS—could reduce fungal virulence and improve crop resistance sustainably. *FoCrpA* also offers biotechnological potential: gene editing (e.g., CRISPR) or plant-derived inhibitors could engineer wilt-resistant crops, while structure-guided antifungal agents may selectively block fungal copper transport with minimal environmental impact. Crucially, *FoCrpA* regulates osmotic/oxidative stress adaptation and infection structures, making it pivotal for both understanding pathogenesis and developing control strategies for rice seedling blight. Future work should validate field applications, explore copper-immune synergies, and refine targeted inhibitors to bridge lab discoveries with agricultural practice.

## Figures and Tables

**Table 1:** Primers used in this study.

Primer Name	Primer Sequence
Hyg-F	TCGCCCTTCCTCCCTTTATTTCAG
Hyg-R	CTACACAGCCATCGGTCCAGAC
SuR-F	CTCTCCGTTGCTTATCCTTGCCTA
SuR-R	CGCCATCACTACGCCTTGTCTT
*FoCrpA*-UP-F	GATCTTCACTAGTGGGAATTCCCCGTGATGATTTGCCCAATGAAT
*FoCrpA*-UP-R	TTGGGTACCGAGCTCGAATTCCGCCATGTTGACTGCTGTGAGA
*FoCrpA*-DN-F	TGGGGATCCTCTAGAGTCGACTGGTATTGGCAGTGGTAGTGTTGG
*FoCrpA*-DN-R	CTTGCATGCCTGCAGGTCGACCCGACGGAGGATCAAGATGTAAGC
Hup-R	TGCTCACCGCCTGGACGACTAA
Hdn-F	TGGACCGATGGCTGTGTAGAAGT
Cup	AGGCTTGGAGGAGAATGGTTGG
Cdn	AAGCCTTCCTTACGCCTGATGATG
*FoCrpA*-F	TGCTCACCGCCTGGACGACTAA
*FoCrpA*-R	TGGACCGATGGCTGTGTAGAAGT
C-*FoCrpA*-F	CCGGGTACCGAGCTCGAATTCCCGTTGCTCTGCCGTATCTTGAA
C-*FoCrpA*-R	AGCTGTCAAACATGAGAATTCAAGCCTTCCTTACGCCTGATGATG
Actin-F	GTTGCCTGAGACTTGACGACGAT
Actin-R	CTCCTCCGAACCATCCGCTACA

**Table 2:** Disease index of the wild-type *F. oxysporum* Fo21 and mutant strains.

Stains	Disease Index
Fo21	74.30 ± 5.11 a
*∆FoCrpA*	48.90 ± 4.85 b
*∆FoCrpA-C*	76.37 ± 3.65 a

Mean ± SD was calculated from the results of three independent experiments. Values on the table followed by the same letter are not significantly different at *p* ≤ 0.01 according to Duncan’s multiple range test. Each experiment was replicated three times.

## Data Availability

The data presented in this study are available on request from the corresponding author.
